# The tip of the iceberg: high-risk contacts for hemorrhagic fevers of swine in the Caribbean

**DOI:** 10.1186/s13567-026-01719-9

**Published:** 2026-02-25

**Authors:** Guillermo Arcega Castillo, Rachael Schulte, Michelle L. Schultze, Luis Pablo Hervé-Claude, B. C. Birtcil, Lisa Boden, Juan Pablo Villanueva-Cabezas, Christa A. Gallagher, María José Navarrete-Talloni, Andres M. Perez, Rachel A. Schambow

**Affiliations:** 1 Center for Animal Health and Food Safety, College of Veterinary Medicine, University of Minnesota, St. Paul, MN 55108 USA; 2 Department of Biomedical Sciences, Ross University School of Veterinary Medicine, Basseterre, Saint Kitts and Nevis; 3 Department of Clinical Sciences, Lewyt College of Veterinary Medicine, Long Island University, Brookville, NY 11548 USA; 4 Royal (Dick) School of Veterinary Studies, University of Edinburgh, Easter Bush Campus, Roslin, EH25 9RG UK; 5 Facultad de Medicina Veterinaria, Universidad San Sebastián, Concepción, Chile; 6 Department of Biomedical Sciences, Lewyt College of Veterinary Medicine, Long Island University, Brookville, NY 11548 USA

**Keywords:** Transboundary animal diseases, commodity flow networks, disease introduction risk, participatory mapping, porcine viral diseases, West Indies

## Abstract

There are major gaps in understanding the transmission risk of hemorrhagic fevers of swine, such as African swine fever (ASF) and classical swine fever (CSF), in the Caribbean because animal-trade data are limited and fragmented. To address this gap, we reconstructed country-level directed trade networks from United Nations Comtrade data for 2022–2024 merged with participant input and analyzed them using social network analysis; nodes represent Caribbean jurisdictions with at least one trade tie, and edges represent reported movements of live pigs or pork products. Using these baselines, we conducted a participatory modeling exercise with 15 veterinary and agricultural officers from 10 Caribbean countries and territories during a regional ASF preparedness workshop in Saint Kitts. Network metrics were estimated for both pig and pork trade networks. Participatory input markedly expanded connectivity: for live pigs, the UN Comtrade network contained 6 edges and experts added 12 (18 total; 67% new). For pork products, the preliminary network contained 58 edges; participants added 42, bringing the total to 100 (42% new). The pork product network’s most central exporters by out-degree were the Dominican Republic (13) and Trinidad and Tobago (12); the median out-degree was 3. The live-pig network remained sparse, with isolated sub-graphs. Despite a median out-degree of 1 across the live-pig network, Saint Kitts and Nevis led exports (out-degree 5), with pig movements to Montserrat, Sint Eustatius, Sint Maarten, Anguilla, and Antigua and Barbuda. These findings show that participatory approaches with local expertise fill data gaps, enhance preparedness, and support risk assessment.

**Supplementary Information:**

The online version contains supplementary material available at 10.1186/s13567-026-01719-9.

## Introduction

African swine fever (ASF) and classical swine fever (CSF) are viral hemorrhagic diseases of swine that cause severe morbidity and mortality in affected swine herds. ASF and CSF outbreaks result in severe economic consequences due to their high mortality and trade restrictions [1–6]. These hemorrhagic fevers are transmitted through a variety of pathways, including contact with infected animals and animal tissues, swill feeding of uncooked pork products, and contaminated fomites like vehicles, equipment, or feed, as both viruses can persist in the environment and in pork products that are chilled or processed with low heat, allowing for transboundary spread [2–4, 7–11]. No effective treatment for either disease has been identified. Accordingly, control relies on prevention: vaccination when available for CSF [12] and biosecurity and movement control for both diseases. There are currently no widely approved ASF vaccines, and research is ongoing [13, 14]. Although vaccines that can differentiate between diseased and vaccinated animals (DIVA) are available for CSF, the most widely used CSF vaccines are modified live vaccines that do not provide DIVA, resulting in trade restrictions in countries that practice vaccination [12, 15]. ASF and CSF are present in large parts of the world, including parts of the Americas. ASF has been identified in ongoing outbreaks in the Dominican Republic and Haiti since 2021 [16, 17], and CSF is considered endemic in some countries and territories [4]. Neighboring pork-producing countries in the region, such as Mexico and the United States, are currently free from both diseases, but incursion of either virus would result in severe economic losses and the potential for the virus to become established in wildlife [3, 5, 11, 18–23]. Two qualitative assessments evaluated the risk of ASF incursion for Caribbean, Central American, and selected South American countries and territories [24, 25]. Both identified eleven to twelve countries and territories with elevated risk via multiple pathways; and one assessment reported insufficient data to assess risk for 24 countries and territories [24, 25].

The Caribbean is a diverse region of over 30 islands, coastal countries, and territories surrounding the Caribbean Sea and situated between North and South America. They are commonly grouped together into the Greater Antilles (Cuba, Jamaica, Hispaniola [Haiti and the Dominican Republic], and Puerto Rico) and the Lesser Antilles (smaller islands) [26]. While these islands and coastal countries are in close geographical proximity, they differ in their culture, sovereignty, geography, policy, and economy. Caribbean countries and territories range in size from the island of Saba, with a population of 2030 and land area of 23 km^2^, to Guyana, with a population of over 831 thousand and a land area of approximately 196 850 km^2^ [27–30]. Within the islands, 19 have a land size of less than 1000 km^2^, often resulting in limited natural resources and heavy reliance on imported goods. Due to these dependencies, logistics and supply chains play a vital role in the region's ability to obtain goods and provisions. For example, Sint Maarten, a Dutch territory, is a central logistics hub for the region. Large cargo vessels will dock, consolidate the cargo into smaller shipments, and then redistribute to the surrounding smaller islands such as Saba, Sint Eustatius, Antigua, Montserrat, and Saint Kitts and Nevis. This system allows for smaller islands without large port capabilities to receive imported materials [31]. Similar to many regions across the globe, the complexity of inter-Caribbean trade and its relevance to disease spread is still poorly understood, limiting the ability for robust risk assessment for spread of diseases like ASF and CSF.

Participatory epidemiology (PE) was developed to address gaps in conventional veterinary epidemiology by eliciting experiential knowledge from stakeholders across the animal health system that is often not captured by traditional epidemiological methods [32]. PE recognizes the value of knowledge held by community members who are often excluded from the research process, engaging those community members in the research and planning process to provide contextual data and to generate policy with their input and knowledge, thereby increasing relevance and successful outcomes [32–34]. Different PE approaches utilize different levels of participation from community stakeholders, employing a variety of methods to generate knowledge, including interviews, participatory mapping, and direct observation [32]. PE has been used to enhance disease surveillance and control programs in many resource-limited settings [32–36], and can be highly valuable when paired with analytical techniques such as network modeling and analysis. Network analysis is a statistical method of evaluating the relationships between defined elements, such as populations or individuals [37, 38]. Each element is a node, and the connections between nodes, called edges, are defined as directed (being unidirectional) or undirected (representing connection without a specific direction) [37, 38]. Network analysis has been increasingly used for various purposes in veterinary medicine, such as to estimate disease transmission risk within and between communities and to identify nodes that present a higher risk for disease spread, ultimately to inform targeted surveillance or intervention strategies [37, 39–42].

As live pig and pork trade are pathways of high potential risk for ASF and CSF transmission, and given that ASF is currently present in the Dominican Republic and Haiti, characterizing inter-Caribbean pig and pork trade will help in estimating regional risk of ASF and CSF spread in the Americas. However, publicly available information in the Caribbean regarding the trade and movement of animals and products is limited. Therefore, the primary objective of this study was to use PE methods to elicit previously unreported inter-Caribbean movements of live pigs and pork products, and then use network analysis to reconstruct directed trade networks for risk relevant characterization. The secondary objective was to compare these participatory inputs with publicly available trade data to quantify the additional connectivity revealed by PE and to discuss implications for ASF/CSF risk in the region.

## Materials and methods

### General approach

Data on live pig and pork trade in the Caribbean were collected using a two-stage, participatory approach, whereby preliminary data were obtained from the United Nations Comtrade database (described below), and then additional data were collected from local experts through a participatory modeling exercise. All countries and territories that are geographically located in the Caribbean and/or considered culturally a part of the Caribbean (i.e., Suriname, Guyana, Venezuela) were included in the scope of this work (see Additional file 1). The scope of trade connections was limited to those in the Caribbean, and outside connections were not considered here. Data on the characteristics of the territories within the Caribbean region were collected using publicly available sources, along with estimated pig population data collected from questionnaires administered to certain territories. These questionnaires are the basis of a related study on the preparedness of countries and territories in the region to respond to and control incursions of ASF [43]. Information about the sovereign status of each territory was collected to show what territories were independent and what territories were otherwise non-self-governing [44]. The estimated swine population was collected from structured interviews in the local language (English, French, or Spanish) with veterinary service representatives from the selected countries and territories as part of a related study [43], or the Food and Agriculture Organization of the United Nations (FAO) database [45]. The human population and current gross domestic product (GDP in US dollars) data for the majority of the territories were obtained from the World Bank database [46] and Census International Databases [47]. Land size (defined as the area under national sovereignty and is the sum of land area, excluding inland waters and coastal waters) was obtained from the FAO database. For territories where information was not available from these sources, others were used [48, 49] and are listed in Additional file 1.

### Preliminary network development

Data were accessed from the United Nations (UN) Comtrade database to build preliminary networks of live pig and pork trade between Caribbean countries and territories based on existing and accessible data [50]. UN Comtrade is a publicly available database of aggregated annual and monthly trade statistics for a wide variety of products and for global trading partners. Data are received from countries and territories, then compiled and standardized by the UN Statistics Division. Products are listed under Harmonized System (HS) codes, a globally-used and standardized nomenclature [51]. HS codes use a hierarchical structure, where groupings from higher levels aggregate all subcategories. Through UN Comtrade, we accessed annual data on imports, exports, foreign imports, and domestic exports from all modes of travel during 2022–2024. This timeframe was chosen because it represents the time period after detection of ASF in Hispaniola, following which trade would have been impacted by various restrictions. Thus, trade data from prior to the ASF outbreaks were excluded. In UN Comtrade, the reporter is the country/territory that submits the trade data, and the partner is the counterpart as declared by the reporter. For imports, the partner corresponds to the country of origin; for exports, the partner corresponds to the country of last known destination. We retrieved records for all available reporters and partners, then filtered to pairs within the Caribbean. UN Comtrade may also include a second partner field, which records the country of consignment (dispatching country for imports; for exports, often the first intermediate country when reported). To maintain consistency and avoid double-counting via consignment routes, we used the primary partner field for origin/destination attribution and set the second-partner field to “World,” so consignments are not counted both under the consignor and the final trading partner, aggregating flows rather than treating consignment paths as separate transactions. This procedure prevents double-counting by ensuring consignments are aggregated to the final trading partner only, rather than being counted once for the consignor and again for the final destination. For network construction, we relabeled the exporting entity as origin and the importing entity as destination. Within UN Comtrade, we extracted live pig trade records classified under HS code 0103 (Swine; live). For pork products, codes relevant to processed and unprocessed pork meat products were included (020641, 020649, 021011, 021012, 021019, 160241, 160242, and 160249; Additional file 2).

UN Comtrade data were downloaded and further processed for network analysis using Microsoft Excel version 2506 and R version 4.4.1 [52, 53]. Data were filtered by selecting only those with trade between Caribbean countries and territories. Notably, Saba and Sint Eustatius (both territories of the Netherlands) are not specified in UN Comtrade data, and Saint Martin (a territory of France) is only listed as a partner. Exporting countries and territories were labeled as “origin”, and importing countries and territories were labeled as “destination” for the networks. Separate directed networks for live pig trade and pork trade were then created and visualized using the igraph package (v1.6.0) in R [54]. Within each network, nodes represented countries and territories, while directed edges represented trade of live pigs or pork products from the origin country to the destination country. To support interpretation of the networks during the participatory exercise, additional figures were created that displayed the volume of trade between countries and territories (Additional file 2; Additional file 3; Additional file 4).

### Participatory modeling

The participatory modeling exercise was conducted as part of a broader educational workshop on ASF preparedness organized by Ross University School of Veterinary Medicine in Saint Kitts from June 24 to 25, 2025. Ross University coordinated the invitations and convened Chief Veterinary Officers (CVOs) and equivalent senior officials representing national agricultural and veterinary authorities from across the Caribbean. These participants were chosen because they occupy key policymaking and enforcement roles within their respective veterinary and agricultural services, including oversight of import permits, inspections, and disease control measures. Through these responsibilities, they maintain direct operational knowledge of live animal and product movements, both formally regulated and informal, within and between islands. Their participation was therefore particularly valuable, as it brought expert insight into the trade dynamics of their territories and aligned with the workshop’s broader goal of strengthening regional coordination and preparedness for ASF. Given their institutional roles and direct engagement in trade regulation and disease control, we considered them highly suitable participants for this activity, as their expertise ensured that the information elicited reflected authoritative, operational knowledge of regional movements. Our research team, in collaboration with Ross University, engaged participants at the event in a structured activity designed to characterize regional trade and movement patterns of live pigs and pork products. The exercise aimed to highlight the importance of trade connections and their relevance as risk pathways for disease transmission. In total, the session included 15 participants from 10 countries and territories, six from Saint Kitts and Nevis and one from each of the following: Trinidad and Tobago, Sint Eustatius, Saint Lucia, Dominican Republic, Suriname, Anguilla, Saba, Montserrat, and the British Virgin Islands. The modeling session was conducted over a two-hour period with six moderators present. Prior to the workshop, all moderators met in person and reviewed the intended format and instructions for the session. To help the participants understand the networks, the nodes within each network were given fixed positions using spatial coordinates such that they were mapped geographically. Then, the live pig network and the pork product network maps were printed as large-format posters (48 inches wide × 36 inches tall; ≈122 × 91 cm; one per participant group per network) for participants to review and interact with.

Before the activity began, moderators explained that the UN Comtrade baseline maps were likely missing informal or unrecorded connections. Participants were explicitly asked to identify any additional routes based on their professional experience, local knowledge, or communication with other veterinary services. To ensure a shared understanding, moderators clarified the definitions of formal and informal trade and provided concrete examples to guide discussion. Formal trade was defined as movements officially recorded through customs declarations, veterinary certificates, or trade statistics, while informal trade referred to unregistered exchanges such as small-scale shipments between islands, transport of pork products by travelers, or undocumented transfers between neighboring territories. Moderators also emphasized that the objective was to capture both types of movements to improve regional understanding of disease risk, and that all individual contributions would remain anonymous to promote honest and open participation.

At the beginning of the session, the participants were introduced to the concept of trade as a potential route of disease introduction. Moderators delivered a presentation on publicly available data regarding formal and registered human and commercial links between countries and territories in the region. Participants were then divided into two groups (hereafter “Group A” and “Group B”), with 8 and 7 participants in Groups A and B, respectively. Participants from Saint Kitts and Nevis, the most represented country, were present in both groups. Each group received an identical set of poster-sized printouts showing the network maps of live pig trade and pork product trade based on publicly available data from UN Comtrade. Participants were instructed to work collaboratively within their groups to review and confirm or correct the connections already marked on the maps, add any missing connections based on their knowledge of both formal and informal trade routes (not limited to their own country), and discuss any relevant country-specific context about how these connections might influence the risk of ASF introduction and spread in the Caribbean. Here, formal trade refers to movements recorded in official customs or trade statistics, whereas informal trade refers to movements outside official reporting channels that are not captured in UN Comtrade or national databases. Throughout the session, moderators helped facilitate discussions, clarified instructions, and supported data capture by typing detailed notes on emerging conversations. Additional context and narrative information shared by participants were also recorded via typed notes to help interpret the network annotations.

The session concluded with a full-group discussion highlighting the importance of acknowledging the extensive and often underrecognized connectivity among Caribbean countries and territories, especially in the context of shared vulnerability to transboundary animal diseases such as ASF and CSF.

After both groups completed their maps, moderators reconciled and consolidated all annotations. A route was included in the final dataset only when a recognized official with direct operational knowledge of formal or informal movements provided a clear and credible justification. Credibility therefore rested on participants’ institutional roles and professional experience; this approach aligns with established best practices for expert knowledge elicitation in data limited settings, where veterinary and agricultural officials often possess operational information not captured in public records [55, 56].

As part of the companion ASF preparedness survey [43], we conducted site visits in selected Caribbean territories to examine import/export manifests, observe container inspections, and document the presence of contraband pork products in local markets. These observations were used qualitatively to contextualize and triangulate workshop findings, not to validate specific edges.

### Social network analysis

The preliminary directed networks were updated to include connections identified following the participatory modeling exercise. To describe the networks, including network structure, connectivity, and centrality of countries and territories, social network analysis was conducted using the igraph package (v1.6.0) in R [54]. Graph-level metrics included network size (nodes and edges), diameter, mean distance, edge density, transitivity (global clustering coefficient), assortativity coefficients, and centralization indices for degree, closeness, and betweenness [57, 58]. Node-level metrics included degree (in and out), closeness centrality (in and out), betweenness centrality, hubs and authority scoring, and eccentricity (in and out) [57, 58]. Metric definitions are summarized in Additional file 5. Additionally, the proportion of new, unique links identified through the PE exercise was calculated.

A community detection algorithm was applied to identify groups of countries and territories with increased trade connections. Community detection refers to the process of identifying groups of nodes within a network that are more densely connected to each other than to nodes outside the group [59]. Here, the infomap community detection algorithm was used [60, 61]. Infomap is particularly well-suited for directed networks and operates based on information theory, aiming to partition nodes into modules that minimize the description length of random walks through the network. This means it detects natural groupings of nodes where flow, such as trade, tends to circulate more frequently within a group than between groups.

Assortativity metrics were calculated to assess the extent to which countries and territories with similar land size, swine population, human population, GDP, and sovereignty status tended to trade with one another [62, 63]. The *assortativity* function in the package *igraph* was used for numeric attributes (land size, swine population, human population, and GDP) and *assortativity_nominal* was used for categorical attributes (sovereignty status, Additional file 1). To evaluate the potential association of geographic closeness and network connectivity, a univariable logistic regression model was used, where the binary outcome was the presence or absence of an edge between two nodes (countries or territories) in the network, and the only predictor was the Euclidean distance between the countries/territories. The Euclidean distance between country/territory centroids (latitude and longitude) was calculated using ArcGIS Pro version 3.5.2 [64]. An odds ratio was estimated by implementing the univariable logistic regression model in R using the glm function [65]. ArcGIS Pro was also used to generate choropleth maps where countries and territories were colored based on their community, as identified via the infomap algorithm.

## Results

### Data reconciliation

Moderators guided the map-based annotation phase to validate existing links, add missing routes, specify directionality, and classify movements as either transshipments or final destinations. Participant differences mainly arose when determining whether certain routes represented direct trade or re-exports routed through larger hubs. Disagreement also occurred regarding whether some movements were ongoing or historical, and whether they involved commercial consignments or small-scale exchanges, such as those carried by travelers. Only connections supported by at least one participant with direct operational knowledge were retained in the dataset.

Reconstruction challenges stemmed from uneven territorial representation, as some islands had multiple officials present, along with uncertainty about routing through intermediary ports and confidentiality concerns when discussing unregistered or informal trade. Given the sensitive nature of these discussions and the confidentiality assurances provided to participants, detailed information about specific routes, actors, or shipment types is not reported here. To address these, we anonymized contributions, labeled movement types, avoided double-counting transshipments, and did not impute volumes or frequencies.

### Network of live pig trade

**Figure 1:**
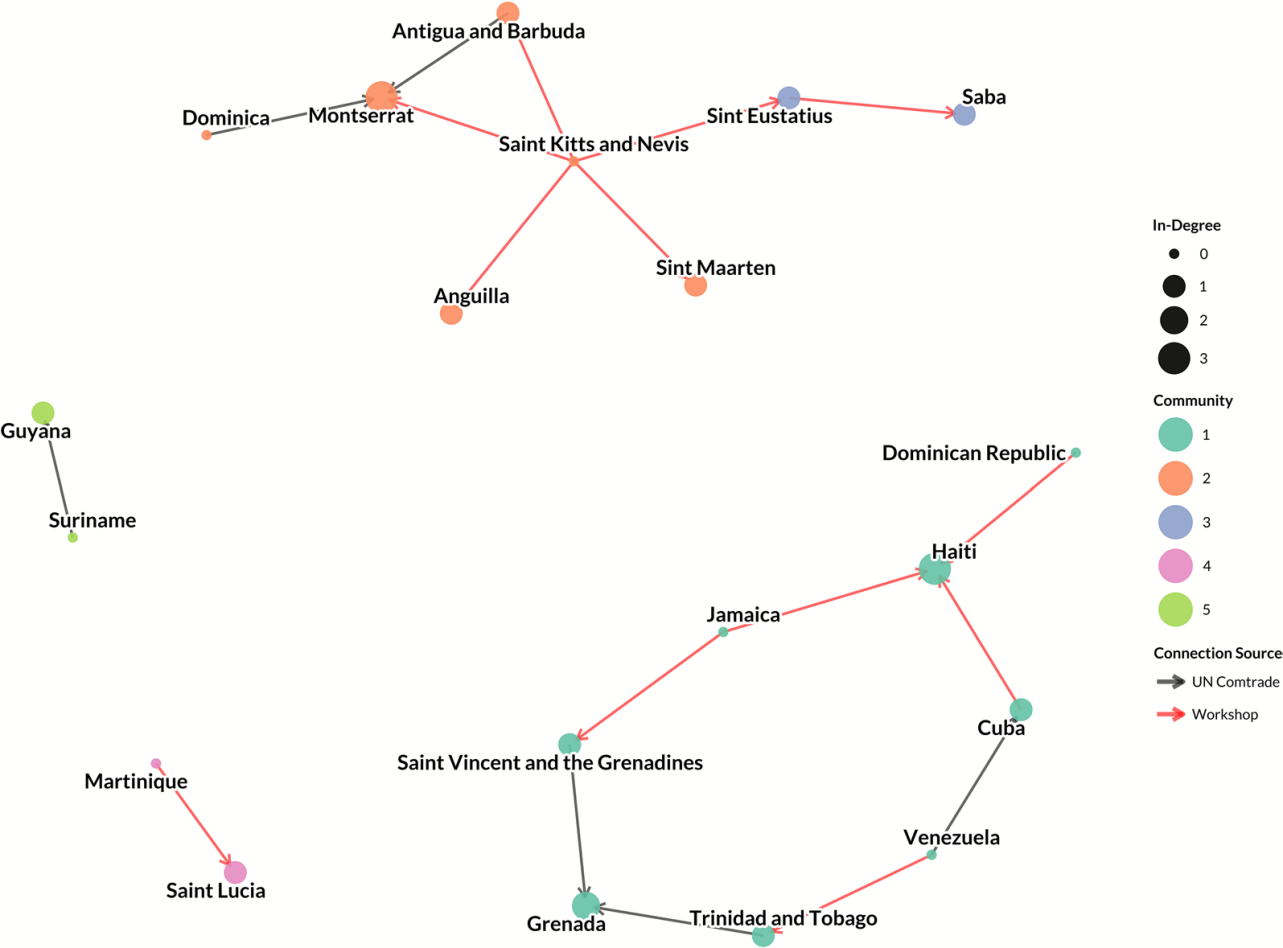
**Network of live pig trade between Caribbean countries and territories, with node size reflecting a country or territory’s in-degree and node color reflecting community.**

**Figure 2:**
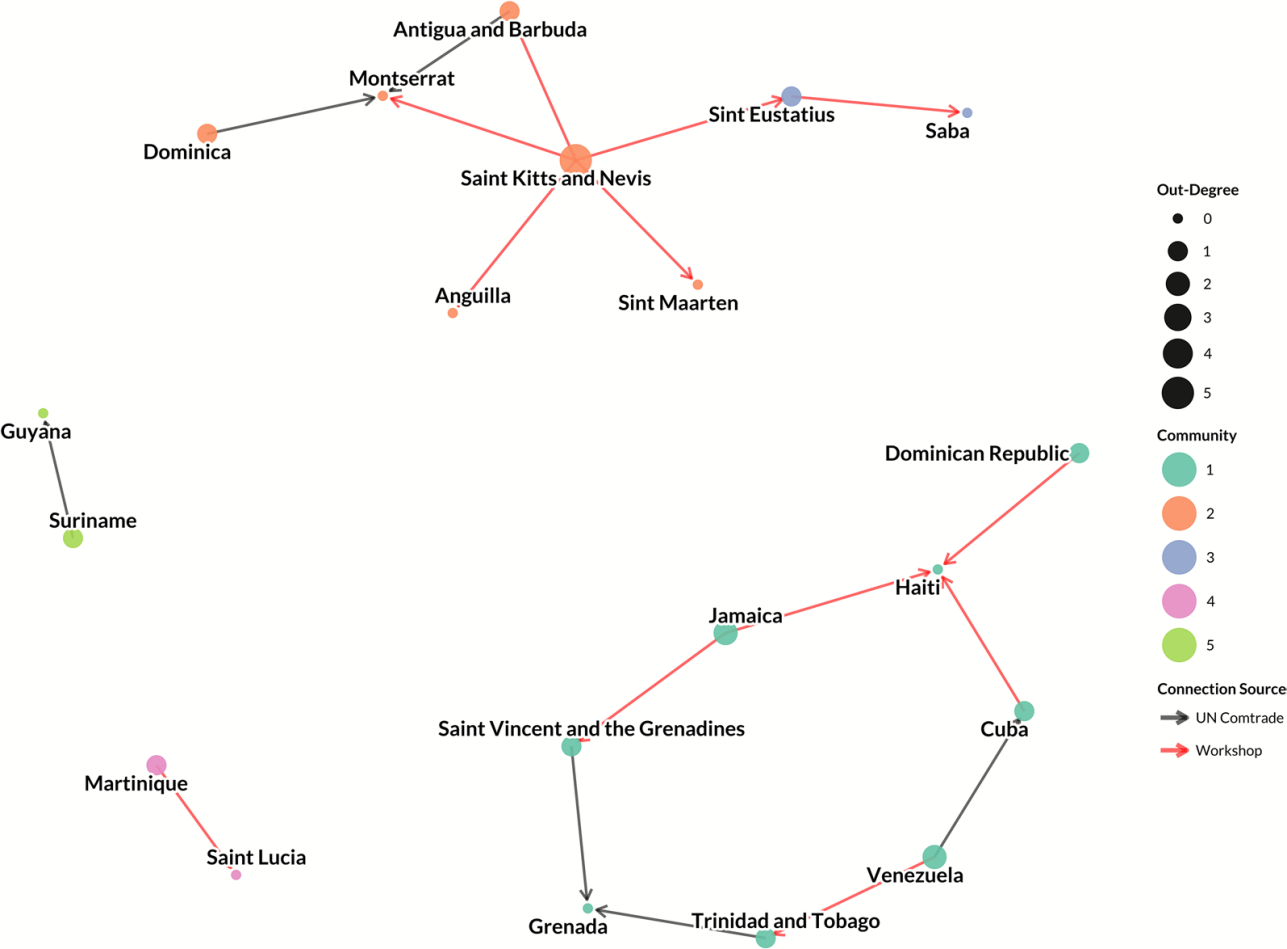
**Network of live pig trade between Caribbean countries and territories, with node size reflecting a country or territory’s out-degree and node color reflecting community.**

The live pig trade network consisted of 20 nodes (countries and territories) and 18 unidirectional edges representing live pig movements (Figures 1 and 2). All countries and territories with reported live pig trade also were present in the pork trade network. Few (*n* = 6, 33%) edges originated from UN Comtrade data, whereas the majority (*n* = 12, 67%) were identified through inputs from workshop participants. Overall, participants reported that most of the connections identified during the workshop were suspected to involve informal movements, rather than formally regulated trade, or may only be documented by one country or territory (exporter or importer).

**Figure 3:**
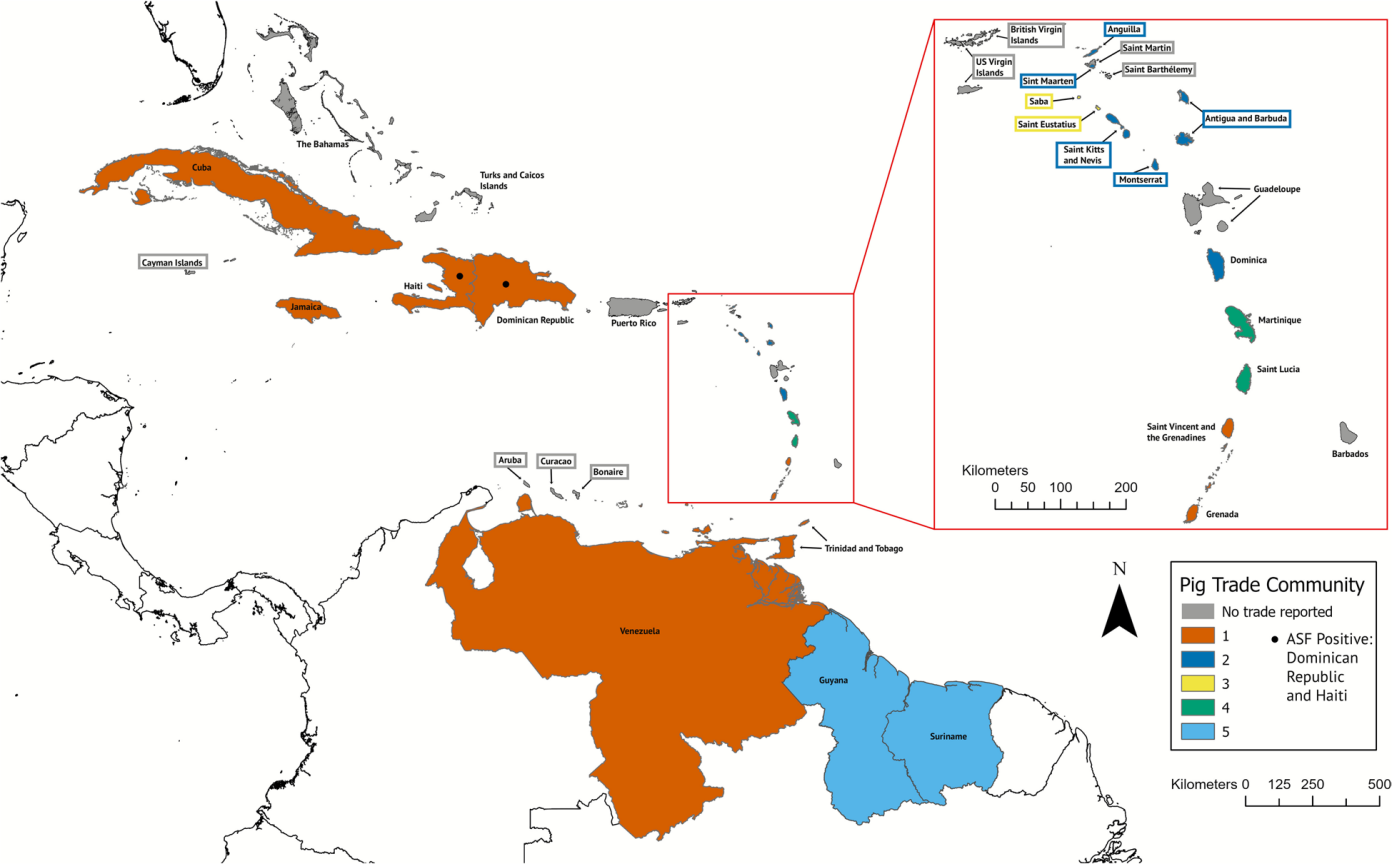
**Caribbean countries and territories by community (color, legend) for live pig trade Countries and territories with gray fill had no reported trade from UN Comtrade or from the participatory input**. Countries with no color fill were not considered in this analysis (non-Caribbean), but were included for geographic context.

The live pig trade network was highly disconnected, with four separate, unconnected sub-graphs present. Two of the larger sub-graphs consisted of eight nodes and eight edges each (Figures 1 and 2). One sub-graph is centered on Saint Kitts and Nevis, which acts as a source node with outbound, unidirectional ties to geographically close islands (Figures 1 and 3). Additionally, there were two sub-graphs with only two nodes and one unidirectional edge each (Martinique to Saint Lucia, and Suriname to Guyana). Because the live pig network is split into four disconnected sub-graphs and is dominated by one-way ties, many node pairs are unreachable. In such directed, disconnected graphs, standard betweenness and closeness centrality metrics cannot be calculated for the network as a whole [66]. We therefore summarize degree and component structure. Nodes with the highest in-degree (corresponding to imports of live pigs) were Montserrat (in-degree: 3), Haiti (in-degree: 3), and Grenada (in-degree: 2; Figure 1; Additional file 6). Seven nodes (Dominica, Dominican Republic, Martinique, Jamaica, Suriname, Saint Kitts and Nevis, and Venezuela) had an in-degree of zero. In terms of out-degree (corresponding to exports of live pigs), Saint Kitts and Nevis was the highest (out-degree: 5), with connectivity to Montserrat, Sint Eustatius, Sint Maarten, Anguilla, and Antigua and Barbuda. Jamaica, Saint Vincent and the Grenadines, and Venezuela all had an out-degree of 2 (Figure 2, Additional file 6). Eight nodes had an out-degree of zero: Montserrat, Grenada, Haiti, Guyana, Saba, Anguilla, Saint Lucia, and Sint Maarten. Reported volume of live pig trade in UN Comtrade data was also low: only five bilateral flows were recorded, totaling ~ 403 pig units (Additional file 3). Three of the five flows were < 15 units (from Antigua and Barbuda to Montserrat: 2 units; from Dominica to Montserrat: 3 units; from Trinidad and Tobago to Grenada: 15 units), with only two flows > 100 units (from Saint Vincent and the Grenadines to Grenada: 263 units; from Suriname to Guyana: 120 units).

Five communities were identified in the live pig network (Figures 1 and 3). Community 1 was one sub-graph which included Trinidad and Tobago, Saint Vincent and the Grenadines, Grenada, Venezuela, Dominican Republic, Cuba, Haiti, and Jamaica. Community 2 consisted of Saint Kitts and Nevis, Antigua and Barbuda, Montserrat, Anguilla, Dominica, and Sint Maarten. Community 3 included Sint Eustatius and Saba. Community 4 and 5 accounted for the two disconnected structures, comprised of Martinique and Saint Lucia (Community 4) and Guyana and Suriname (Community 5). Nodes were highly assortative for GDP (assortativity: 0.80), moderately assortative for human population (assortativity: 0.53), swine population (assortativity: 0.44), and land size (assortativity: 0.37), and less assortative for sovereignty (assortativity: 0.13). Countries and territories that were geographically farther from each other were also significantly less likely to have an edge present between them (OR = 0.8, 95% CI 0.70–0.91, *p* < 0.05).

### Network of pork product trade

**Figure 4:**
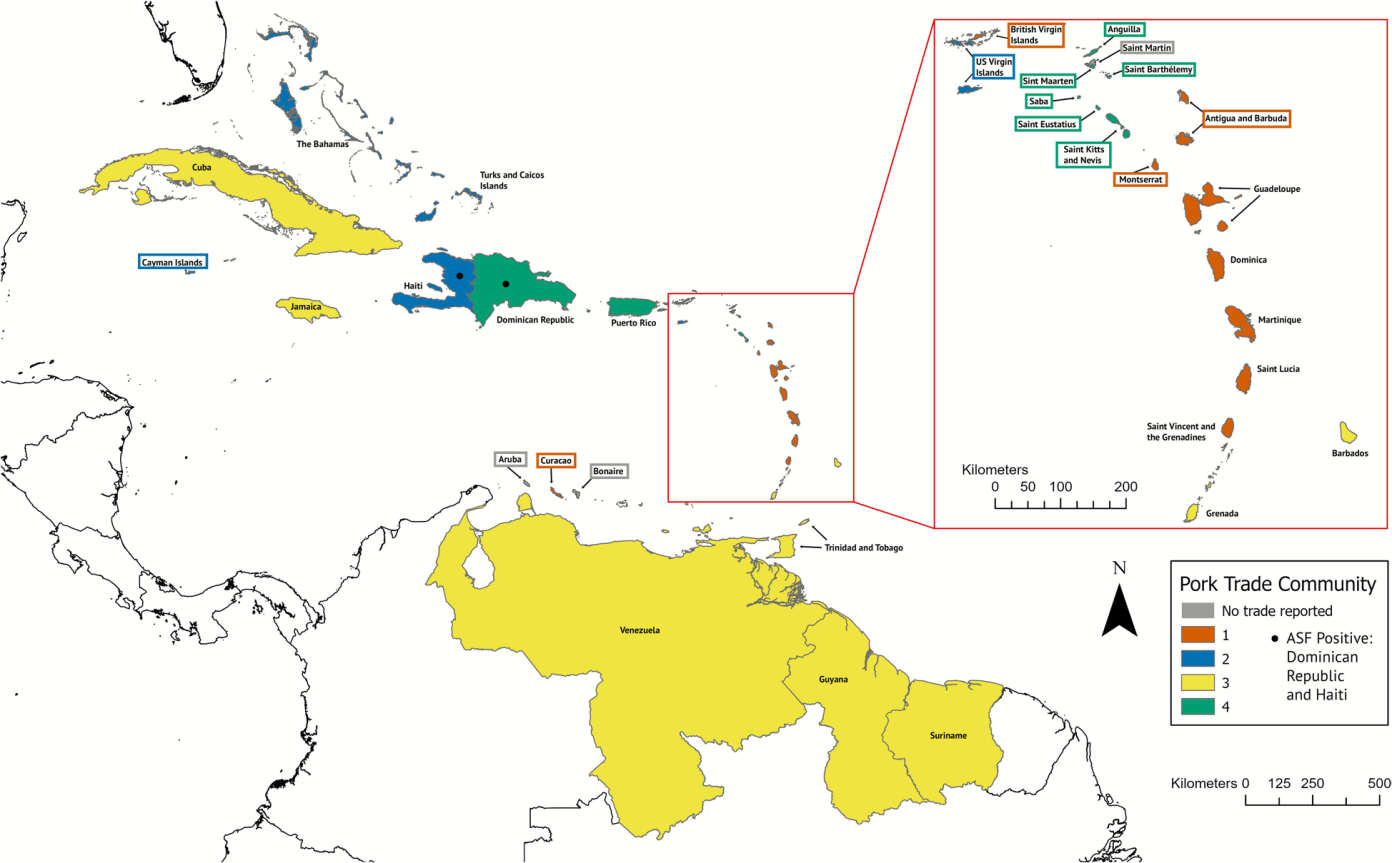
**Caribbean countries and territories by community (color, legend) for pork product trade**. Countries and territories with gray fill had no reported trade from UN Comtrade or from the participatory input. Countries with no color fill were not considered in this analysis (non-Caribbean), but were included for geographic context.

**Figure 5:**
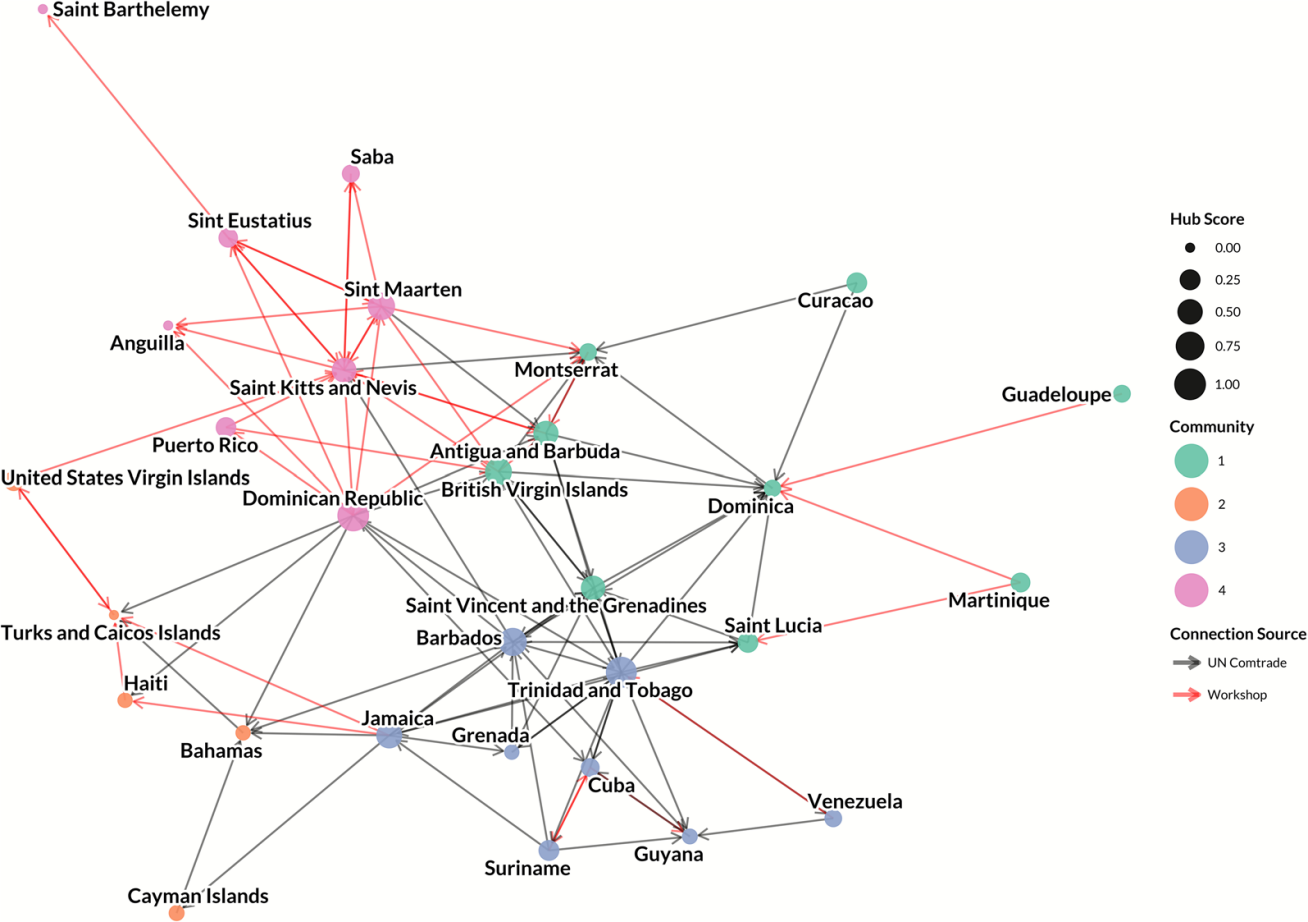
**Network of pork product trade between Caribbean countries and territories, with node size reflecting hub score and node color reflecting community membership.**

**Figure 6:**
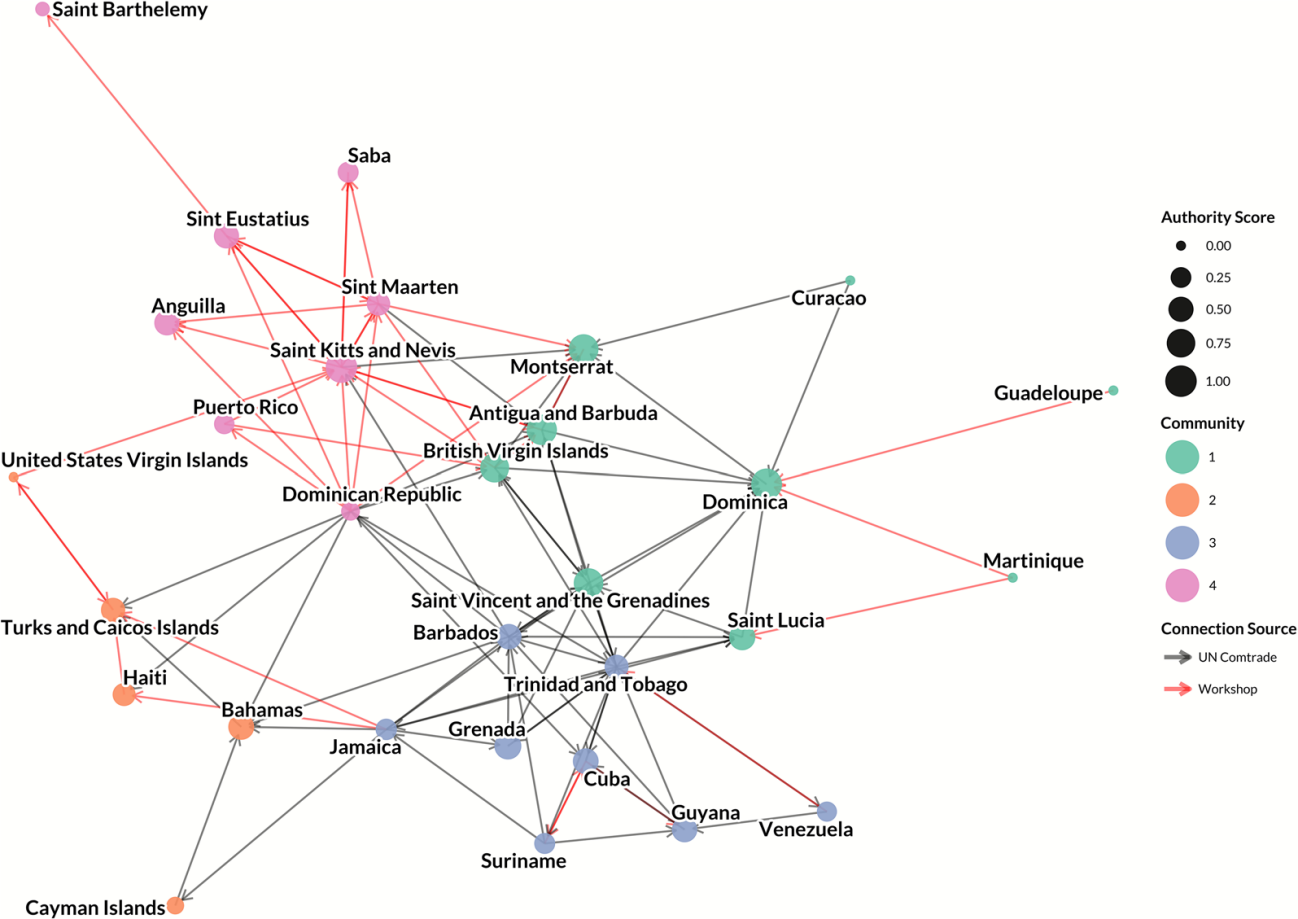
**Network of pork product trade between Caribbean countries and territories, with node size reflecting authority score and node color reflecting community membership.**

The pork trade network has 50% more nodes and approximately 5.6 times as many edges as the live pig network (Figures 4, 5, and 6). The pork trade network consisted of 30 nodes and 100 edges. Of these, 58 unique edges were from UN Comtrade data and 42 (42%) were reported by workshop participants. Participant-reported connections included both formal trade and undocumented movements. All nodes present in the live pig network were present in the pork product network, and additional nodes were present in the pork product network: the Bahamas, Barbados, British Virgin Islands, Cayman Islands, Curaçao, Guadeloupe, Puerto Rico, Saint Barthélemy, Turks and Caicos Islands, and United States Virgin Islands. The average degree was 6.66 (median in-degree = 3, median out-degree = 2; Additional file 7), the network diameter was 9, the mean distance of shortest paths was 3.45, and edge density was 0.11. Global transitivity was 0.42. Global centralization metrics were low to moderate, with betweenness centralization slightly higher (0.3) than degree centralization (0.18). As previously described, the live pig network consisted of several disconnected components, so we focused on measures that apply to disconnected graphs [66], while the pork product network allowed for a fuller set of connectivity metrics.

Nodes with the highest in-degree were Saint Kitts and Nevis (in-degree: 9), Dominica (in-degree: 9), and Montserrat (in-degree: 7; Additional file 8). Nodes with the highest out-degree were the Dominican Republic (out-degree: 13), Trinidad and Tobago (out-degree: 12), Jamaica (out-degree: 8) and Barbados (out-degree: 8). The majority of countries and territories had relatively few exports, with 18 of the 30 nodes having an out-degree of 2 or less. In-closeness centrality was highest amongst Antigua and Barbuda, Montserrat, Dominica, and Saint Kitts and Nevis (all have an in-closeness centrality of 0.44), whereas out-closeness centrality was highest amongst the Dominican Republic (out-closeness: 0.65), Trinidad and Tobago (out-closeness: 0.54), Barbados (out-closeness: 0.51), and Jamaica (out-closeness: 0.46). Betweenness centrality was highest for Saint Vincent and the Grenadines (betweenness: 0.36), Antigua and Barbuda (betweenness: 0.30), Trinidad and Tobago (betweenness: 0.28), and Saint Kitts and Nevis (betweenness: 0.28). The Cayman Islands, Guadeloupe, Haiti, Curaçao, Saba, Martinique, Guyana, Puerto Rico, Grenada, Venezuela, Anguilla, and Saint Barthélemy all recorded a betweenness centrality of 0, and had in-closeness ranging from 0.20 to 0.32 and out-closeness ranging from 0.16 to 0.36, with some values undefined due to reachability. Consistent with this, the highest out-eccentricity values were observed in the Cayman Islands (out-eccentricity: 9), Guadeloupe, the Bahamas, and Haiti (each with an out-eccentricity of 8), and the highest in-eccentricity values were Puerto Rico and Haiti (in-eccentricity: 9). Hub scores were highest for the Dominican Republic (hub score: 1.00), Trinidad and Tobago (hub score: 0.93), and Barbados (hub score: 0.70, Figure 5). The lowest hub scores were recorded in Anguilla, Saint Barthélemy, and the Turks and Caicos Islands (all hub scores of 0). Authority was highest for Saint Kitts and Nevis (authority score: 1.00) followed closely by Dominica (authority score: 0.95), and Antigua and Barbuda (authority score: 0.90) (Figure 6). Curaçao, Guadeloupe, Martinique, and the United States Virgin Islands all had authority scores of 0.

HS codes associated with pork trade from UN Comtrade data were mainly meat preparations (offal, hams) and fresh, chilled, or frozen pork (Additional file 2). Based on the UN Comtrade data alone, where individual export and import movements were available (Additional file 4), Trinidad and Tobago had by far the highest volume of exports (1 620 573 kg), followed by Jamaica (859 352 kg), Barbados (327 890 kg), and the Dominican Republic (223 373 kg). The Dominican Republic exported to seven different partners (Antigua and Barbuda, Bahamas, British Virgin Islands, Cuba, Haiti, Trinidad and Tobago, and Turks and Caicos Islands), the majority of which were to Trinidad and Tobago (121 519 kg) and the Bahamas (94 085 kg).

Four communities were identified within the pork product network (Figure 4). Community 1 included Antigua and Barbuda, British Virgin Islands, Curaçao, Dominica, Guadeloupe, Martinique, Montserrat, Saint Lucia, and Saint Vincent and the Grenadines. Community 2 included the Bahamas, the Cayman Islands, Haiti, the Turks and Caicos Islands, and the United States Virgin Islands. Community 3 included Barbados, Cuba, Grenada, Guyana, Jamaica, Suriname, Trinidad and Tobago, and Venezuela. Community 4 included Anguilla, the Dominican Republic, Puerto Rico, Saba, Saint Barthélemy, Saint Kitts and Nevis, Sint Eustatius, and Sint Maarten. Assortativity was low for land area (assortativity: 0.15), swine population (assortativity: -0.02), human population (assortativity: 0.06), GDP (assortativity: 0.07), and sovereignty status (assortativity: 0.09), though countries and territories that were geographically farther away were significantly less likely to have an edge present between them (OR = 0.90, 95% CI 0.87–0.94, *p* < 0.05).

## Discussion

This study represents one of the first assessments of pig and pork product trade connectivity between countries and territories in the Caribbean region. Because data on live pig and pork trade are often limited in many regions of the world and informal trade is not officially reported, this work used a participatory approach to identify formal and informal trade movements of live pigs and pork products. These findings revealed many previously publicly unknown trade connections, which is highly important given ASF’s ongoing presence in Haiti and the Dominican Republic.

Overall, the live pig network was relatively small (20 nodes, 18 edges) and disconnected even after participatory input (four disconnected sub-graphs), with several isolated nodes and few long-range paths (Figure 1). Consistent with this observation, pig trade reported by UN Comtrade was limited (Additional file 3). By contrast, continental pig trade networks are orders of magnitude larger and substantially more connected. For example, national analyses of Germany’s pig trade system report approximately 100 000 premises and hundreds of thousands of trade links, conditions under which a single introduction can traverse large portions of the network [67]. These benchmarks suggest that, compared with such high connectivity systems, the Caribbean live pig network’s small size, fragmentation, and predominantly one-way ties imply limited capacity for sustained within-region spread following an introduction. Findings from a comparable island network in Mayotte, a French overseas department in the Indian Ocean, show that in small, fragmented systems (a 17 node livestock movement network) transmission concentrates through a few bridging hubs [68]. This mirrors our Caribbean live pig network, where limited connectivity and one-way ties suggest any within-region spread would be funneled through a small set of high centrality nodes (for example, Saint Kitts and Nevis). Taken together, these comparisons support a risk profile in which introduction risk into the Caribbean (from extra-regional sources or via higher-connectivity pork product pathways) is the dominant concern, whereas extensive spread via live-pig movements within the region appears comparatively constrained under current connectivity.

However, the potential impact on transmission risk remains a concern because routine pre-movement diagnostic testing is uncommon; testing is generally limited to outbreak movement permits and regional analysis [25, 69, 70]. Additionally, risk reduction can be pursued through swill-feeding restrictions, targeted surveillance, movement permitting and traceability, and improved biosecurity [69, 71]. As highlighted during the participatory modeling workshop and previous questionnaire interviews, there is a lack of accessible and timely diagnostic tools and laboratory infrastructure across the region, particularly for detecting transboundary diseases such as ASF and CSF, which results in considerable time delays and logistical complexity [43]. In this context, point-of-care (POC) tests could be especially valuable by enabling rapid, field-level screening of pigs prior to movement, even in remote or resource-limited areas, thereby enhancing early detection and reducing the risk of transboundary disease spread [72, 73]. If employed, POC testing should be conducted under protocols established by a competent authority using validated assays, trained operators, and quality assurance and quality control procedures with mandatory reporting [74]. As this is a relatively new technology, its implementation should be preceded by careful evaluation of when and where its use is most appropriate, and it should operate under a clear regulatory framework to ensure reliability, proper oversight, and consistency with existing surveillance systems. It should serve as a triage step within movement-permitting frameworks, with any positive or non-negative results confirmed by an accredited laboratory (e.g., PCR) before movement.

Across the Caribbean, mitigation commonly includes veterinary import permits and certification, border inspections and seizures, movement controls such as quarantine, standstills and zoning, swill-feeding bans, farm biosecurity and cleaning and disinfection, traceability and movement permitting, surveillance in passive, risk-based and slaughter settings, and prevention and contingency plans under veterinary legislation [43]. Our companion survey finds these frameworks exist but implementation is uneven, with major gaps in backyard and feral population characterization, risk-based and wildlife surveillance, traceability, laboratory surge capacity, staffing and funding, traveler communications, and regular simulation exercises, with the largest shortfalls in island territories [43].

In comparison to the live pig trade network (20 nodes, 18 edges), the pork product network, both formally reported and undocumented, had 30 nodes and 100 edges (1.5 × as many nodes; 5.6 × as many edges; Figures 1, 2, 5, and 6). Top exporters by out-degree were the Dominican Republic (13), Trinidad and Tobago (12), and Jamaica (8); top importers were Dominica (9), Saint Kitts and Nevis (9), and Montserrat (7). Consistent with this, the largest commercial pork producers in the region are Cuba (approximately 214 000 metric tons), the Dominican Republic (more than 85 000 metric tons), and Haiti (approximately 31 000 metric tons); whereas Trinidad and Tobago, despite its role as a pork product exporter, produces approximately 1900 metric tons commercially [75].

Community structure, which reflects groups of countries and territories with increased trade, appeared to be influenced by both geographic proximity and administrative and historical affiliations (Figures 3 and 4, Additional file 1). In the live pig network, Saint Kitts and Nevis serves as the central exporter to surrounding territories, many of which are either British Overseas Territories or members of the Commonwealth of Nations [76]. Community 3 included the Dutch territories of Sint Eustatius and Saba, community 4 linked the neighboring islands of Martinique and Saint Lucia, and community 5 comprised the neighboring South American countries of Suriname and Guyana. The pork product network showed similar patterns, as communities 1, 2, and 4 generally included countries and territories that were geographically close to each other. Both live pig and pork product trade also had networks (Community 1 for live pigs, and Community 3 for pork products) that were more geographically dispersed in the Caribbean and mainland South America. Both communities included Trinidad and Tobago, Grenada, Venezuela, Cuba, and Jamaica. Beyond geography, political–economic alliances can also shape connectivity; for example, Venezuela and Cuba maintain long-standing preferential bilateral ties (e.g., subsidized oil-for-services arrangements and coordinated cooperation frameworks), which may facilitate trade links and help explain their co-membership in these communities [77]. Other real-world factors may greatly influence community structure, such as the size of ports and accessibility for large vessels, which has been previously reported; for example, Sint Eustatius receives the majority of its imports from Sint Maarten, as the port’s infrastructure (lack of deep seaports) is not capable of handling larger container ships [31]; and workshop participants from other small islands, such as Saba and Anguilla, reported similar restrictions.

Because participant input revealed undocumented export pathways and concentrated bridging roles not captured in public trade statistics, the resulting network structure is directly relevant to risk assessment. Accordingly, the trade connections identified here have potentially important implications for ASF and CSF introduction and spread within the region and, by extension, across the Americas. Despite the Dominican Republic’s known ASF-positive status, both UN Comtrade data and participants reported trade of pork products from the Dominican Republic to 13 other Caribbean countries and territories. Notably, only seven trading partners with the Dominican Republic were identified through UN Comtrade data alone (Additional file 4). It should be noted, however, that the types of pork products exported are not specifically identified by UN Comtrade, and may include some movements for non-trade reasons, such as tissue specimens for disease diagnosis. However, during the participatory workshop, participants reported many informal movements from the Dominican Republic, such as trade of processed salami, that were not available via publicly accessible data. While the number of pork exports from the Dominican Republic was relatively small, the informal nature of this trade suggests that the risk may be more significant than would be estimated if publicly available trade data were considered alone. However, although these within-region pathways exist, ASF has not yet been detected in other Caribbean countries or territories receiving these exports. This may be due to multiple reasons: many products mentioned are cooked/processed in ways that reduce infectivity, reported volumes appear limited/episodic, pig populations in importing countries are often small (Additional file 1), several territories restrict swill feeding [43], and controls from the Dominican Republic might prevent significant export of pork products from sick pigs. Together, these considerations may explain the absence of the detected spread of ASF to date. Nevertheless, because the full network of trade remains unknown, the cumulative risk remains highly uncertain and cannot be reliably assessed with the available data. While we did not compare within-region and extra-regional risks, phylogenetic and quantitative assessments indicate that extra-regional introductions, particularly via passenger-carried pork products, constitute a substantial risk pathway [11, 78], and the 2021 Hispaniola strain clusters within the Georgia 2007 genotype II lineage [79]. Therefore, although this work focuses on intra-regional trade risk, extra-regional pathways cannot be ignored. UN Comtrade indicates that many Caribbean territories import pork products primarily from the United States and European suppliers, and territories with constitutional ties to European states often procure through their administering states [43, 50]. Our companion preparedness survey likewise noted frequent air and sea links with North America and Europe, dependence on regional transshipment hubs, and traveler carriage of meat products, with enforcement and traveler communications varying across jurisdictions [24, 43].

The participatory approach used here reveals the limitations of using only publicly available data for risk assessment, particularly where formal data capture and reporting capacity is low. Overall, 46% of the trade connections identified here were collected from participatory input. For instance, the high representation of participants from Saint Kitts and Nevis enabled the identification of detailed live pig trade connections involving that territory, most of which were absent from official UN Comtrade records. This example underscores the critical role of local stakeholder engagement in uncovering informal or underreported trade pathways and serves as a model for future exercises to further characterize trade in the region. At a broader scale, omitting these data from models could lead to erroneous conclusions and suboptimal disease mitigation strategies. Additionally, PE approaches such as this educate and raise awareness of disease risks amongst veterinary stakeholders, which can increase their prioritization and ownership of ASF and CSF preparedness and prevention. Conducting comparable PE exercises across additional Caribbean communities and territories would help complete the regional trade map, reduce uncertainty around informal pathways, and better support the design of sustainable, country-specific mitigation strategies.

Several limitations should be considered when interpreting the findings of this study. Publicly available data from UN Comtrade did not include individual reports for Saba and Sint Eustatius, which may be reported under broader administrative categories (e.g., the Netherlands). HS codes report trade products at relatively broad levels, and the nature of the actual product moved is not known. Additionally, intermediary stops and/or the final destination of products is not always reported or clear from trade data. The participatory modeling exercise included 15 participants from 10 countries and territories, of which 6 were from Saint Kitts and Nevis. This introduced a geographical bias, with overrepresentation of trade dynamics involving Saint Kitts and Nevis and underrepresentation of countries and territories that were not present. For example, the Dominican Republic had an in-degree of zero in the presented network though informal trade of pork and pigs has been speculated from Haiti [80]; participation in the activity by a Haitian representative may have led to further elucidation of the directionality of this trade. This exemplifies the need to continue participatory data collection in the region, and future participatory studies should aim to include participants from more Caribbean countries and territories. Also, the accuracy and completeness of data collected by participatory approach relies on participants’ recall, experience, and willingness to share information. While moderators facilitated structured discussions and encouraged participants to contribute information beyond their home countries and territories, important informal or sensitive trade movements may have been underreported, especially if participants were hesitant to share information that could be perceived as illicit [80]; in-depth reporting of trade direction and volume was not captured. Although these participants hold policymaking and enforcement roles, it is acknowledged that they may not be aware of every movement occurring within or between islands. However, those with direct involvement in unregulated or informal trade are typically outside official systems and thus difficult or impossible to reach through structured data collection. As a result, engaging these senior officials represents the most feasible and reliable approach to obtain authoritative information on regional trade patterns. Finally, participant-reported data did not include the volume or frequency of trade movements, limiting the current analysis’ ability to assess the weight or risk level of specific pathways. We acknowledge that key data gaps remain for a fully quantitative risk assessment in the region. However, this study’s primary objective was not to produce an exhaustive census of all trade, but to elicit unreported links via participatory methods and reconstruct and characterize the resulting networks. We also compared the elicited links with UN Comtrade records; that contrast shows how much connectivity and potential risk can be missed when analyses rely solely on formal trade statistics. For those reasons, despite efforts made to fill information gaps and the novelty of our work, arguably, the results here reflect only the “tip of the iceberg” in terms of connectivity of the region.

In conclusion, this study demonstrates the complexities of contacts in the region and limitations of relying solely on formal and publicly available trade records. By integrating participatory modeling with local veterinary and agricultural officers, extensive informal and unregulated movements of live pigs and pork products were found, many of which involved countries and territories with no previously reported connections or were connections from ASF-positive countries. This approach provides a replicable framework for filling critical data gaps in regions with limited formal reporting systems and highlights the importance of leveraging local expertise to improve the accuracy of disease risk assessments. Future work should aim to use participatory epidemiological approaches to collect more complete data on other countries and territories and other aspects of ASF and CSF risk in the Caribbean, such as considering introduction risks from outside the region and collecting additional data on product types, volumes, and frequency of movements. Given the relatively small scale of PE efforts and prevention strategies such as POC tests compared with the costs and consequences of ASF/CSF outbreaks, expanding them across the region is a high-leverage strategy—closing data gaps now will reduce the risk of larger-scale events and reduce overall response costs.

## Supplementary Information

**Additional file 1 Characteristics of Caribbean countries and territories.**

**Additional file 2 Kilograms of pork trade reported to UN Comtrade (imports or exports) for each pork product type by Harmonized System (HS) code between Caribbean countries and territories from 2022-2024.**

**Additional file 3 Volume of live pig trade as reported to UN Comtrade in pig units (imports or exports) between Caribbean countries and territories from 2022-2024 by exporting country (x-axis) and to importing country (legend, colored bars).**

**Additional file 4 Volume (kg) of pork product trade reported to UN Comtrade between Caribbean countries and territories from 2022-2024 by exporting country (x-axis) and to importing country (legend, colored bars), for countries with a) over 20 000 kg of trade reported and b) countries with less than 20 000 kg of trade reported**. Separate panels are used only to improve visualization.

**Additional file 5 Descriptive network metrics for social network analysis, their definition, and interpretation.**

**Additional file 6 Node-level metrics for social network analysis of live pig trade in the Caribbean from 2022-2024.**

**Additional file 7 Boxplots of in- and out-degree distributions for pork trade in the Caribbean from 2022–2024.**

**Additional file 8 Node-level metrics for social network analysis of pork trade in the Caribbean from 2022-2024.**

## Data Availability

The dataset(s) supporting the conclusions of this article are available in the UN Comtrade repository [50]. Data generated during the study for the social network analysis are included within the article and its additional files.
