# Supplementary material for: The tip of the iceberg: high-risk contacts for hemorrhagic fevers of swine in the Caribbean
Source: Vet Res. 2026 Feb 25;57:44. doi: 10.1186/s13567-026-01719-9 (PMC13041270; doi:10.1186/s13567-026-01719-9)
Supplement: Supplementary file 1 — Additional file 1 Characteristics of Caribbean countries and territories. [file 13567_2026_1719_MOESM1_ESM.docx]

Additional file 1: Supplementary Table 1. Characteristics of Caribbean countries and territories.

| **Country / Territory** | **Sovereignty** | **Land Area (Square km)** | **Estimated Total Swine Population** | **Estimated Human Population (Thousands)** | **GDP (Current USD, Millions)** |
| --- | --- | --- | --- | --- | --- |
| Anguilla | United Kingdom | 90^30^ | 75-80^43^ | 19^47^ | 453^46^ |
| Antigua and Barbuda | Sovereign | 440^30^ | 5,126^45^ | 94^46^ | 2,225^46^ |
| Aruba | Kingdom of the Netherlands | 180^45^ | Not Available | 108^46^ | 3,649^f46^ |
| Bahamas, The | Sovereign | 10,010^45^ | 5,024^45^ | 401^46^ | 15,833^46^ |
| Barbados | Sovereign | 430^45^ | 23,505^45^ | 282^46^ | 7,165^46^ |
| Belize | Sovereign | 22,810^45^ | 44,638^43^ | 417^46^ | 3,516^46^ |
| Bermuda | United Kingdom | 50^30^ | Not Available | 65^46^ | 8,980^46^ |
| Bonaire | Kingdom of the Netherlands | 288^80^ | Not Available | 25^f27^ | 3,845^46*^ |
| British Virgin Islands | United Kingdom | 150^45^ | 1,500^b45^ | 39^46^ | 1,597^46^ |
| Cayman Islands | United Kingdom | 240^45^ | 400^b45^ | 74^46^ | 7,139^f46^ |
| Cuba | Sovereign | 106,440^30^ | 1,738,569^45^ | 10,980^46^ | 10,7351^d46^ |
| Curacao | Kingdom of the Netherlands | 444^45^ | Not Available | 156^46^ | 3,281^f46^ |
| Dominica | Sovereign | 750^45^ | 5,072^45^ | 66^46^ | 689^46^ |
| Dominican Republic | Sovereign | 48,320^30^ | 490,000^43^ | 11,428^46^ | 124,676^46^ |
| French Guiana | France | 82,200^30^ | Not Available | 300^80^ | Not Available |
| Grenada | Sovereign | 340^45^ | 4,783^45^ | 117^46^ | 1,391^46^ |
| Guadeloupe | France | 1,620^45^ | 15,128^79^ | 376^80^ | 9,243^79^ |
| Guyana | Sovereign | 196,850^30^ | 9,548^45^ | 831^46^ | 24,836^46^ |
| Haiti | Sovereign | 27,750^30^ | 1,012,016^45^ | 11,773^46^ | 25,224^46^ |
| Jamaica | Sovereign | 10,830^45^ | 218,431^45^ | 2,839^46^ | 19,930^46^ |
| Martinique | France | 1,060^45^ | 20,000^b45^ | 345^80^ | Not Available |
| Montserrat | United Kingdom | 100^45^ | 200^43^ | 5^43^ | 80^f46^ |
| Puerto Rico | United States | 8,870^45^ | 41,557^45^ | 3,203^46^ | 125,842^46^ |
| Saba | Kingdom of the Netherlands | 13^80^ | 50-60^43^ | 2^f27^ | 3,845^46*^ |
| Saint Barthelemy | France | 21^30^ | Not Available | 7^47^ | Not Available |
| Saint Kitts and Nevis | Sovereign | 260^45^ | 679^43^ and 1,800^43^ | 47^46^ | 1,067^46^ |
| Saint Lucia | Sovereign | 610^45^ | 10,835^45^ | 180^46^ | 2,549^46^ |
| Saint Martin | France | 53^30^ | Not Available | 26^46^ | 1,735^46^ |
| Sint Eustatius | Kingdom of the Netherlands | 21^80^ | 100^43^ | 3^f27^ | 3,845^46*^ |
| Sint Maarten | Kingdom of the Netherlands | 34^45^ | <40^43^ | 43^46^ | 649^46^ |
| St. Vincent and the Grenadines | Sovereign | 389^30^ | 6,096^45^ | 101^46^ | 1,157^46^ |
| Suriname | Sovereign | 156,000^30^ | 33,488^45^ | 634^46^ | 4,714^46^ |
| Trinidad and Tobago | Sovereign | 5,130^45^ | 28,376^45^ | 1,368^46^ | 26,429^46^ |
| Turks and Caicos Islands | United Kingdom | 948^30^ | Not Available | 47^46^ | 1,745^46^ |
| United States Virgin Islands | United States | 347^30^ | 2,650^45^ | 104^47^ | 4,670^46^ |
| Venezuela | Sovereign | 882,050^45^ | 25,546,030^45^ | 28,406^46^ | 482,359^a46^ |

^a^2014, ^b^2016, ^c^2017, ^d^2020, ^e^2022, ^f^2023
*Reported as Netherlands Antilles
