# Supplementary figures and images for: The tip of the iceberg: high-risk contacts for hemorrhagic fevers of swine in the Caribbean
Source: Vet Res. 2026 Feb 25;57:44. doi: 10.1186/s13567-026-01719-9 (PMC13041270; doi:10.1186/s13567-026-01719-9)

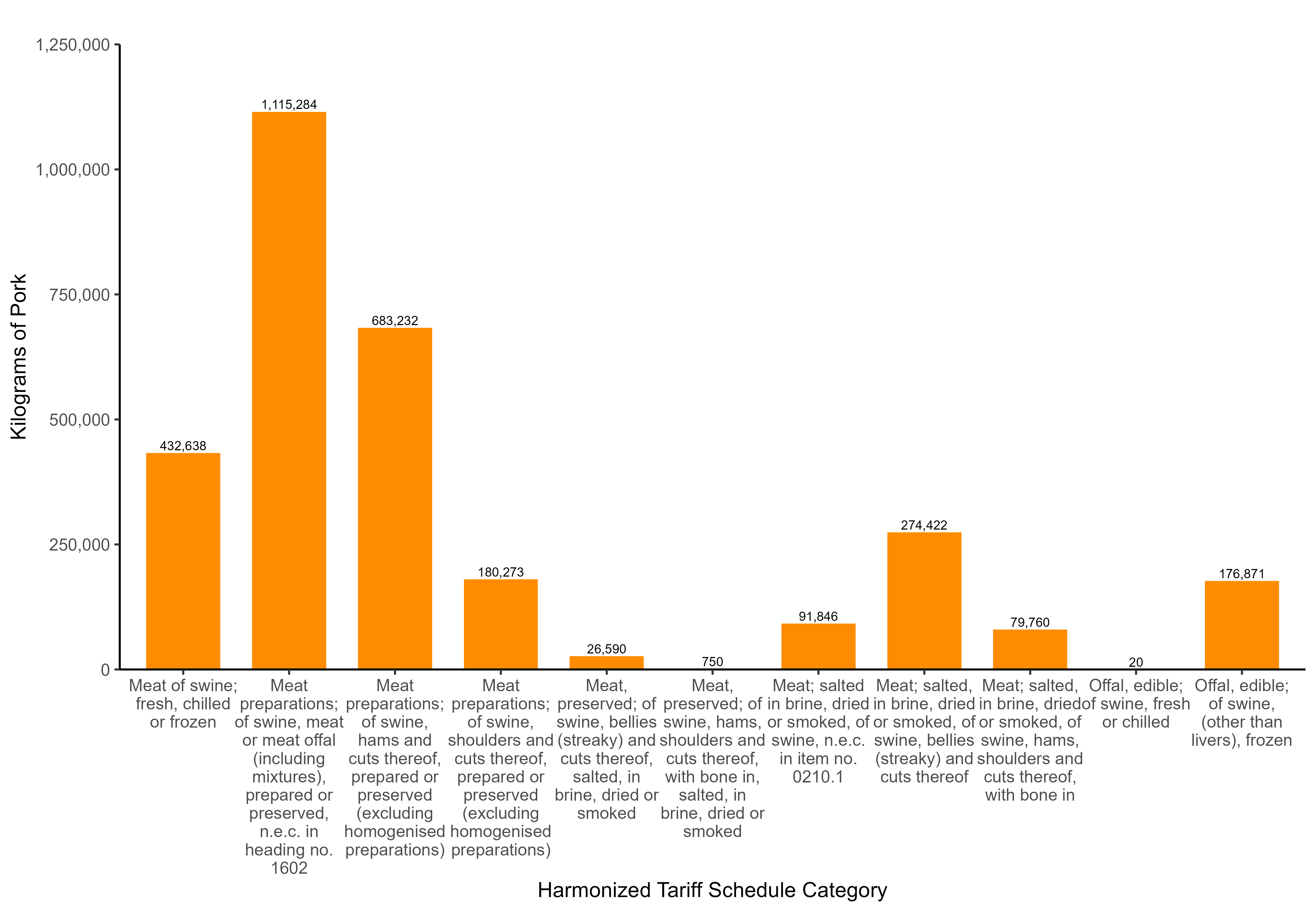

Supplement: Supplementary file 2 — Additional file 2 Kilograms of pork trade reported to UN Comtrade (imports or exports) for each pork product type by Harmonized System (HS) code between Caribbean countries and territories from 2022-2024. [file 13567_2026_1719_MOESM2_ESM.png]

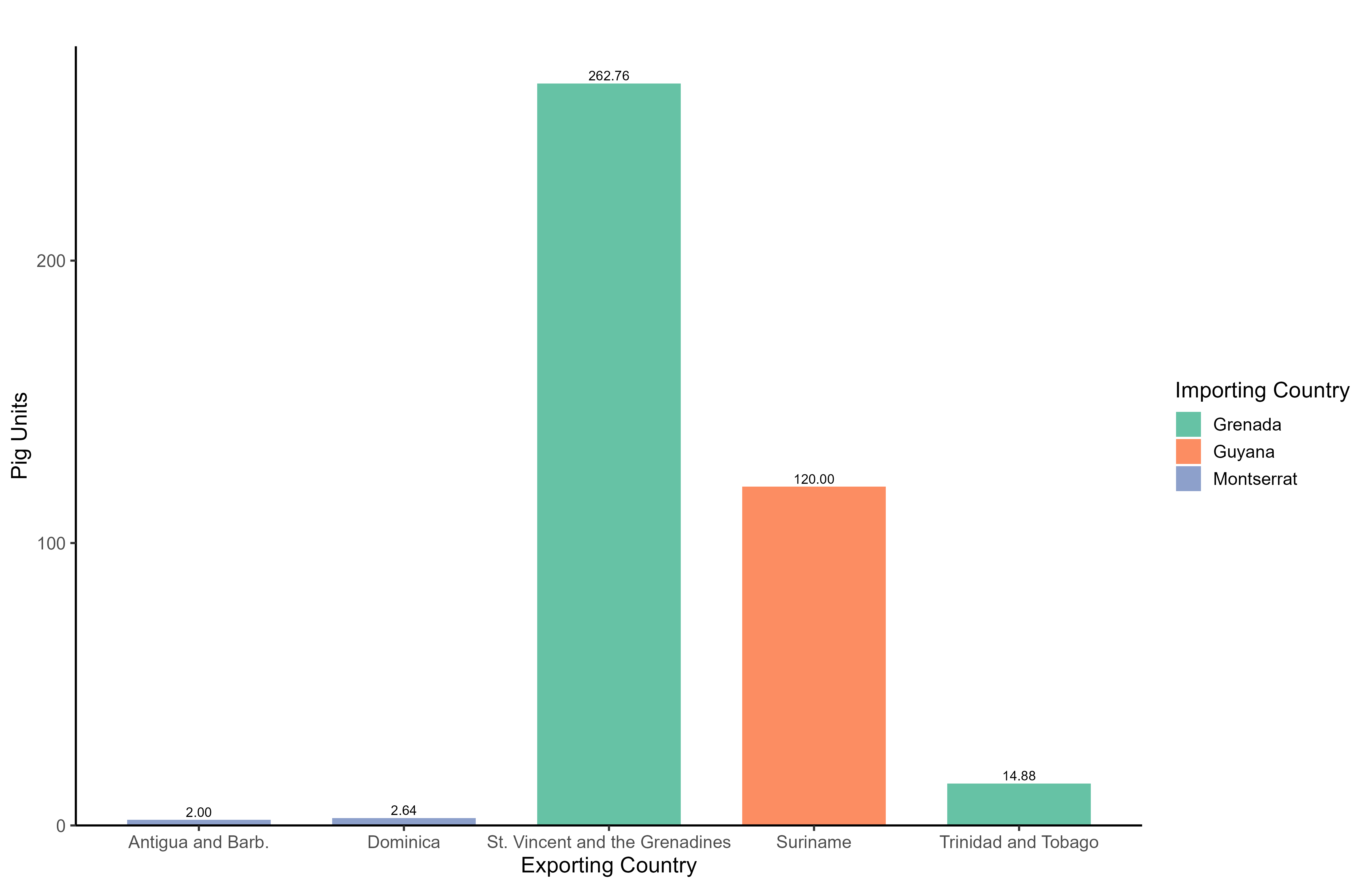

Supplement: Supplementary file 3 — Additional file 3 Volume of live pig trade as reported to UN Comtrade in pig units (imports or exports) between Caribbean countries and territories from 2022-2024 by exporting country (x-axis) and to importing country (legend, colored bars). [file 13567_2026_1719_MOESM3_ESM.png]

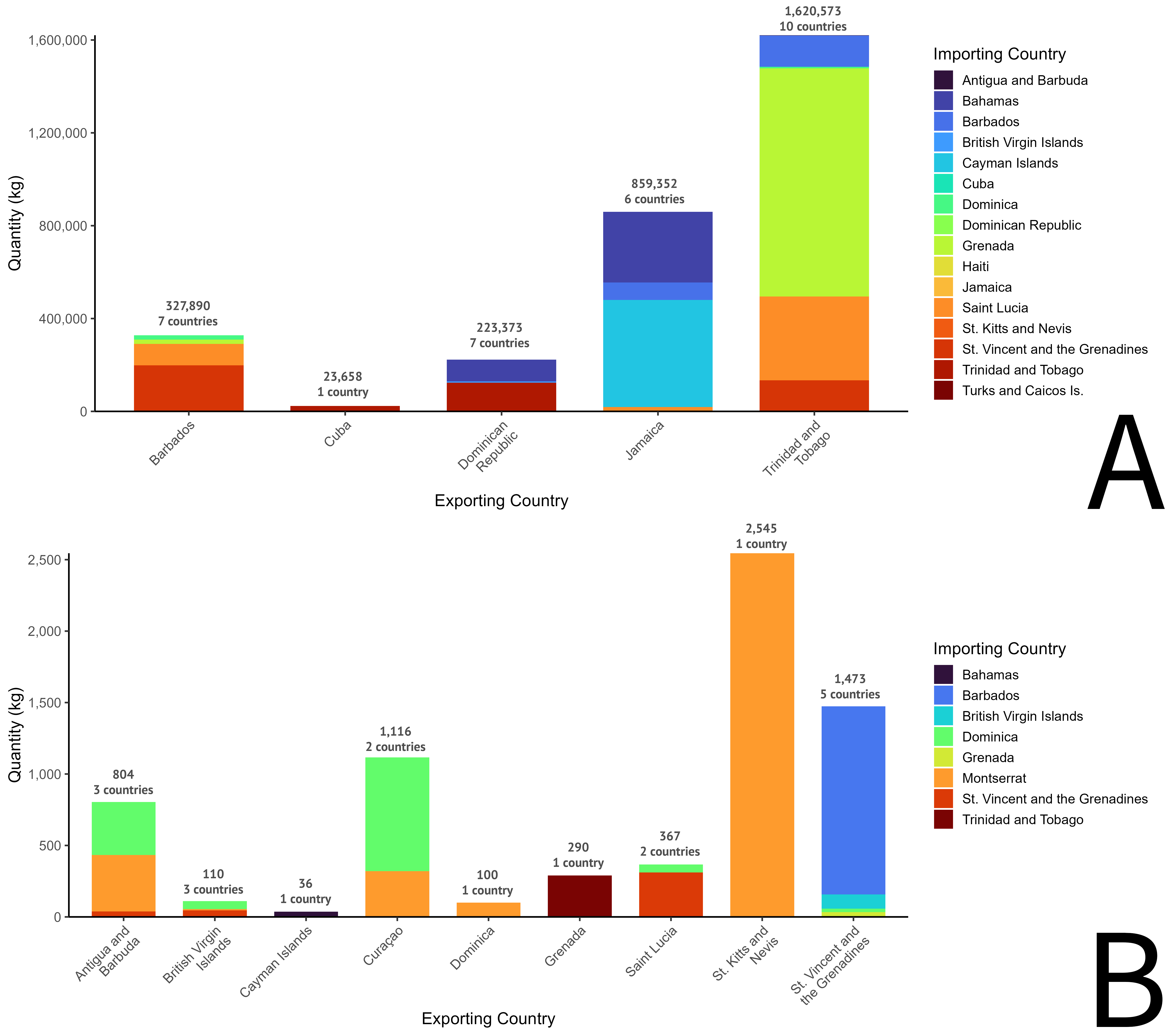

Supplement: Supplementary file 4 — Additional file 4 Volume (kg) of pork product trade reported to UN Comtrade between Caribbean countries and territories from 2022-2024 by exporting country (x-axis) and to importing country (legend, colored bars), for countries with a) over 20 000 kg of trade reported and b) countries with less than 20 000 kg of trade reported. Separate panels are used only to improve visualization. [file 13567_2026_1719_MOESM4_ESM.png]

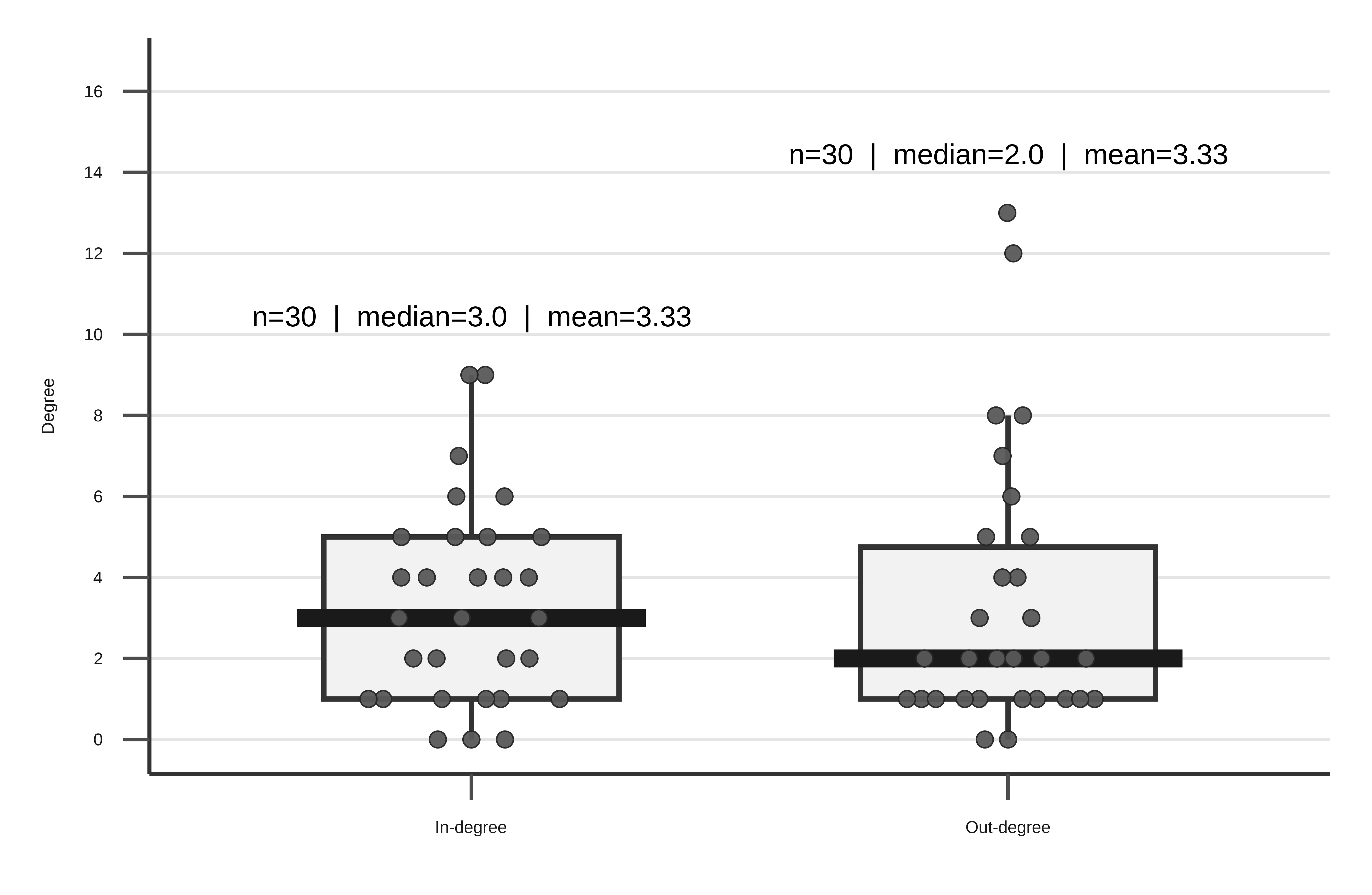

Supplement: Supplementary file 7 — Additional file 7 Boxplots of in- and out-degree distributions for pork trade in the Caribbean from 2022–2024. [file 13567_2026_1719_MOESM7_ESM.png]
