# Supplementary material for: The tip of the iceberg: high-risk contacts for hemorrhagic fevers of swine in the Caribbean
Source: Vet Res. 2026 Feb 25;57:44. doi: 10.1186/s13567-026-01719-9 (PMC13041270; doi:10.1186/s13567-026-01719-9)
Supplement: Supplementary file 5 — Additional file 5 Descriptive network metrics for social network analysis, their definition, and interpretation. [file 13567_2026_1719_MOESM5_ESM.docx]

Additional file 5: Supplementary Table 2. Descriptive network metrics for social network analysis, their definition, and interpretation.

| **Metric Name** | **Definition** | **Interpretation / Value** |
| --- | --- | --- |
| Nodes | Total number of unique nodes in the network. | Positive integer ≥ 1 |
| Edges | Total number of connections between nodes. | Positive integer ≥ 0 |
| Diameter | Longest shortest path in the network. | Positive integers ≥ 1 |
| Mean Distance | Average shortest path length among connected nodes. | Positive integers ≥ 0  High value: nodes are generally distant. Low value: nodes are generally close. |
| Edge Density | Proportion of existing edges out of all possible edges. | 0 (no edges) to 1 (all possible edges present). |
| Degree | Number of edges a node has. In directed networks, in-degree refers to incoming edges, and out-degree refers to outgoing edges. | Positive integer ≥ 0  High value: highly connected node. Low value: isolated node. |
| Closeness | Average shortest distance from a node to all others. In directed networks, may be estimated using incoming paths (in-closeness) and outgoing paths (out-closeness). | 0 (distant to other nodes) to 1 (very close to other nodes). |
| Betweenness | Frequency that a node appears on shortest paths between other pairs of nodes in a network. | 0 (on no shortest paths) to 1 (lies on most amount of shortest paths). |
| Eccentricity | Longest shortest path from or to a node. May be estimated considering only paths to the node (in-eccentricity) or paths from the node (out-eccentricity). | Positive integers ≥ 1  High value: peripheral, distant node. Low value: central, accessible node. |
| Community | Groupings based on dense internal connections. | Categorical labels indicating community. Nodes in the same community: densely interconnected. Nodes in different communities: sparse connections. |
| Global Transitivity (Clustering Coefficient) | Probability neighbors of a node are interconnected, estimated as the ratio of the count of triangles and connected triples in the graph. Edge directions are ignored. | 0 (no clustering) to 1 (complete clustering). |
| Degree Centralization | Degree inequality across the network. | 0 (evenly distributed) to 1 (one node dominates). |
| Betweenness Centralization | Inequality in control of shortest paths. | 0 (evenly distributed) to 1 (one node dominates). |
| Closeness Centralization | Inequality in ease of reaching others. | 0 (evenly distributed) to 1 (one node dominates reachability). |
| Hub | A hub is a node that connects to high-quality or authoritative nodes. May not hold content, but guides network flow. | 0 (few connections to authorities) to 1 (many connections to authorities). |
| Authority | An authority is a node that is frequently pointed to by strong hubs. | 0 (rarely linked to by hubs) to 1 (frequently linked to by hubs). |
| Assortativity | The tendency for nodes to have an edge with nodes with similar characteristics (assortative) or dissimilar characteristics (disassortative). | Continuous variables: −1 to 1, with 1 indicating perfect assortativity and −1 indicating perfect disassortativity.  Categorical variables: 1 indicates perfect assortativity, and 0 indicates no assortative mixing. Dissortative networks lie in the range of -1 to 0, though -1 does not indicate a perfectly dissortative network as with continuous variables. |
