# Supplementary material for: The tip of the iceberg: high-risk contacts for hemorrhagic fevers of swine in the Caribbean
Source: Vet Res. 2026 Feb 25;57:44. doi: 10.1186/s13567-026-01719-9 (PMC13041270; doi:10.1186/s13567-026-01719-9)
Supplement: Supplementary file 6 — Additional file 6 Node-level metrics for social network analysis of live pig trade in the Caribbean from 2022-2024. [file 13567_2026_1719_MOESM6_ESM.docx]

Additional file 6: Supplementary Table 3. Node-level metrics for social network analysis of live pig trade in the Caribbean from 2022-2024.

| **Country / Territory (Node)** | **In Degree** | **Out Degree** |
| --- | --- | --- |
| Anguilla | 1 | 0 |
| Antigua and Barbuda | 1 | 1 |
| Cuba | 1 | 1 |
| Dominica | 0 | 1 |
| Dominican Republic | 0 | 1 |
| Grenada | 3 | 0 |
| Guyana | 1 | 0 |
| Haiti | 3 | 0 |
| Jamaica | 0 | 2 |
| Martinique | 0 | 1 |
| Montserrat | 3 | 0 |
| Saba | 1 | 0 |
| Saint Kitts and Nevis | 0 | 5 |
| Saint Lucia | 1 | 0 |
| Saint Vincent and the Grenadines | 1 | 2 |
| Sint Eustatius | 1 | 1 |
| Sint Maarten | 1 | 0 |
| Suriname | 0 | 1 |
| Trinidad and Tobago | 1 | 1 |
| Venezuela | 0 | 2 |
