# Supplementary material for: The tip of the iceberg: high-risk contacts for hemorrhagic fevers of swine in the Caribbean
Source: Vet Res. 2026 Feb 25;57:44. doi: 10.1186/s13567-026-01719-9 (PMC13041270; doi:10.1186/s13567-026-01719-9)
Supplement: Supplementary file 8 — Additional file 8 Node-level metrics for social network analysis of pork trade in the Caribbean from 2022-2024. [file 13567_2026_1719_MOESM8_ESM.docx]

Additional file 8: Supplementary Table 4. Node-level metrics for social network analysis of pork trade in the Caribbean from 2022-2024.

| **Country / Territory (Node)** | **In Degree** | **Out Degree** | **In Closeness** | **Out Closeness** | **Betweenness** | **In Eccentricity** | **Out Eccentricity** | **Community** | **Hub** | **Authorities** |
| --- | --- | --- | --- | --- | --- | --- | --- | --- | --- | --- |
| Anguilla | 3 | 0 | 0.32 | NC | 0 | 5 | 0 | 4 | 0 | 0.5 |
| Antigua and Barbuda | 6 | 4 | 0.44 | 0.38 | 0.3 | 5 | 5 | 1 | 0.49 | 0.9 |
| Bahamas | 4 | 1 | 0.28 | 0.18 | 0.04 | 7 | 8 | 2 | 0.06 | 0.55 |
| Barbados | 4 | 8 | 0.32 | 0.51 | 0.2 | 7 | 4 | 3 | 0.7 | 0.52 |
| British Virgin Islands | 5 | 5 | 0.36 | 0.39 | 0.06 | 6 | 5 | 1 | 0.61 | 0.78 |
| Cayman Islands | 1 | 1 | 0.22 | 0.16 | 0 | 8 | 9 | 2 | 0.07 | 0.13 |
| Cuba | 4 | 3 | 0.26 | 0.37 | 0.04 | 8 | 5 | 3 | 0.16 | 0.54 |
| Curacao | 0 | 2 | NC | 0.23 | 0 | 0 | 7 | 1 | 0.24 | 0 |
| Dominica | 9 | 1 | 0.44 | 0.22 | 0.04 | 6 | 7 | 1 | 0.11 | 0.95 |
| Dominican Republic | 1 | 13 | 0.25 | 0.65 | 0.08 | 8 | 3 | 4 | 1 | 0.17 |
| Grenada | 4 | 1 | 0.32 | 0.36 | 0 | 7 | 5 | 3 | 0.06 | 0.62 |
| Guadeloupe | 0 | 1 | NC | 0.19 | 0 | 0 | 8 | 1 | 0.13 | 0 |
| Guyana | 5 | 1 | 0.27 | 0.27 | 0 | 8 | 6 | 3 | 0.07 | 0.52 |
| Haiti | 2 | 1 | 0.22 | 0.19 | 0 | 9 | 8 | 2 | 0.06 | 0.37 |
| Jamaica | 2 | 8 | 0.25 | 0.46 | 0.08 | 8 | 4 | 3 | 0.54 | 0.28 |
| Martinique | 0 | 2 | NC | 0.26 | 0 | 0 | 6 | 1 | 0.2 | 0 |
| Montserrat | 7 | 1 | 0.44 | 0.28 | 0.1 | 5 | 6 | 1 | 0.12 | 0.85 |
| Puerto Rico | 1 | 2 | 0.2 | 0.31 | 0 | 9 | 6 | 4 | 0.24 | 0.24 |
| Saba | 2 | 1 | 0.31 | 0.25 | 0 | 5 | 7 | 4 | 0.13 | 0.26 |
| Saint Barthelemy | 1 | 0 | 0.25 | NC | 0 | 6 | 0 | 4 | 0 | 0.05 |
| Saint Kitts and Nevis | 9 | 6 | 0.44 | 0.32 | 0.28 | 4 | 6 | 4 | 0.45 | 1 |
| Saint Lucia | 4 | 2 | 0.27 | 0.32 | 0.03 | 8 | 5 | 1 | 0.24 | 0.57 |
| Saint Vincent and the Grenadines | 6 | 5 | 0.4 | 0.44 | 0.36 | 6 | 4 | 1 | 0.44 | 0.84 |
| Sint Eustatius | 3 | 3 | 0.32 | 0.25 | 0.03 | 5 | 7 | 4 | 0.19 | 0.5 |
| Sint Maarten | 3 | 7 | 0.32 | 0.33 | 0.03 | 5 | 6 | 4 | 0.64 | 0.39 |
| Suriname | 2 | 4 | 0.25 | 0.43 | 0.02 | 8 | 4 | 3 | 0.25 | 0.26 |
| Trinidad and Tobago | 5 | 12 | 0.33 | 0.54 | 0.28 | 7 | 4 | 3 | 0.93 | 0.43 |
| Turks and Caicos Islands | 5 | 1 | 0.28 | 0.21 | 0.12 | 7 | 7 | 2 | 0 | 0.44 |
| United States Virgin Islands | 1 | 2 | 0.22 | 0.27 | 0.12 | 8 | 6 | 2 | 0.19 | 0 |
| Venezuela | 1 | 2 | 0.25 | 0.36 | 0 | 8 | 5 | 3 | 0.13 | 0.22 |
